# Dynamic changes in the transcriptome of tRNA-derived small RNAs related with fat metabolism

**DOI:** 10.1038/s41597-023-02624-y

**Published:** 2023-10-14

**Authors:** Tianci Liao, Mailin Gan, Yuhang Lei, Yan Wang, Lei Chen, Linyuan Shen, Li Zhu

**Affiliations:** 1 Farm Animal Genetic Resources Exploration and Innovation Key Laboratory of Sichuan Province, Sichuan Agricultural University, Chengdu, 611130 China; 2 Key Laboratory of Livestock and Poultry Multi-omics, Ministry of Agriculture and Rural Affairs, College of Animal and Technology, Sichuan Agricultural University, Chengdu, 611130 China

**Keywords:** Small RNAs, RNA sequencing

## Abstract

The prevalence of obesity and overweight is steadily rising, posing a significant global challenge for humanity. The fundamental cause of obesity and overweight lies in the abnormal accumulation of adipose tissue. While numerous regulatory factors related to fat deposition have been identified in previous studies, a considerable number of regulatory mechanisms remain unknown. tRNA-derived small RNAs (tsRNAs), a novel class of non-coding RNAs, have emerged as significant regulators in various biological processes. In this study, we obtained small RNA sequencing data from subcutaneous white adipose tissue and omental white adipose tissue of lean and obese pigs. In addition, we similarly obtained tsRNAs profiles from scapular brown adipose tissue (BAT), inguinal white adipose tissue (iWAT) and epigonadal white adipose tissue (eWAT) of normal mice. Finally, we successfully identified a large number of expressed tsRNAs in each tissue type and identified tsRNAs conserved in different adipose tissues of pigs and mice. These datasets will be a valuable resource for elucidating the epigenetic mechanisms of fat deposition.

## Background & Summary

Adipose tissue plays a crucial role in the regulation of the endocrine system and maintaining energy homeostasis in mammals^1^. The deposition and distribution of adipose tissue are closely linked to obesity-related diseases, including type II diabetes, cardiovascular disease, and metabolic syndrome^2,3^. Fat deposition is a highly intricate biological process influenced by complex interactions between genetics, epigenetics, and environmental factors, and is accompanied by alterations in cell structure and function^4^. While researchers have identified numerous key functional genes, such as PPARγ^5^ and C/EBPα^6^, involved in the regulation of fat deposition, much remains unknown regarding the epigenetic regulatory mechanisms operating at the pre- and post-transcriptional levels.

In recent years, a growing body of research has highlighted the crucial regulatory role of non-coding RNAs (ncRNAs) in various biological processes^7^. Among them, tRNA-derived small RNAs (tsRNAs) have emerged as a newly discovered class of ncRNAs derived from tRNAs^8^. tsRNAs are widely present in a variety of organisms and exhibit functionally regulated ncRNAs with a wide range of biological functions, including protein translation inhibition^9^, regulation of ribosome biogenesis^10^, control of mRNA stability^11^, regulation of cell proliferation^12^, regulation of apoptosis^13^, immune regulation^14^ and epigenetic regulation^15^. tsRNAs can be categorized into two main types: tRNA halves (tiRNAs) and tRNA-derived fragments (tRFs). Based on their corresponding parental tRNA sequence positions and lengths, tsRNAs can be further classified as follows: (i) tRF-5, originating from the 5′ end of mature tRNA and further divided into tRF-5a, tRF-5b, and tRF-5c based on their length; (ii) tRF-3, derived from the 3′ end of mature tRNA and further subdivided into tRF-3a and tRF-3b; (iii) tRF-1, originating from the 3′ end of precursor tRNA; and (iv) tRF-2, corresponding to an intermediate fragment of mature tRNA. tsRNAs are widely distributed across organisms and play crucial regulatory roles in lipid deposition biology^16^. For instance, in mice with nonalcoholic steatohepatitis, blueberry monomeric TEC has been shown to inhibit lipid damage and deposition by promoting tRF-47^17^. Sperm tsRNAs have been implicated as potential mediators for the transmission of maternal high-fat diet-induced addictive behaviors and obesogenic phenotypes to offspring^18^. In high-fat diet mice, elevated levels of 5′tsRNA-Gly-GCC in mature sperm have been found to promote hepatic gluconeogenesis through the regulation of the Sirt6-FoxO1 pathway, resulting in significant weight gain in F0 mice^19^. These pieces of evidence underscore the significant regulatory roles of tsRNAs in fat deposition.

**Fig. 1:**
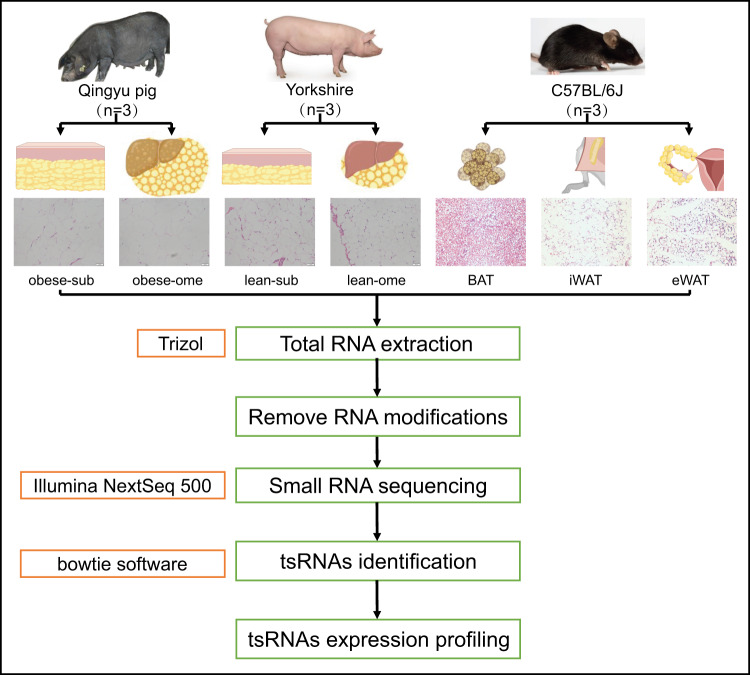
Illustration of sampled animals, sample collection, sample sectioning and RNA extraction process, small RNA sequencing and data analysis. Scale: 1 bar represents 50 μm.

Currently, pigs and mice are the primary animal models used in human medicine. Pigs, being one of the most widely reared livestock species globally^20^, not only provide a significant source of protein for human consumption but also serve as valuable biological models for studying various human diseases^21^. This is due to their similarities to humans in terms of cardiovascular system and metabolic characteristics^22^. Mice, on the other hand, are also extensively utilized as biological models for studying human diseases. They possess a high degree of genetic similarity to humans, exhibit excellent reproductive performance, and are relatively inexpensive to breed and maintain^23^. However, the biological model of the pig has limitations when it comes to studying the biology of fat deposition. The reason is that brown fat does not exist in pigs. The main function of brown fat is thermogenesis, which is mainly useful in human infancy, but some brown fat remains in the adult body^24^. And unlike pigs, brown fat is present in mice. So to investigate the role of tsRNAs in fat deposition, we collected subcutaneous white adipose tissue and omental white adipose tissue from both Qingyu pig (obese pig) and Yorkshine (lean pig) and obtained their tsRNAs sequencing data. In addition, we also obtained tsRNAs sequencing data of scapular brown adipose tissue (BAT), inguinal white adipose tissue (iWAT) and epigonadal white adipose tissue (eWAT) from normal C57BL/6j mice (Fig. 1). Detailed information about the samples can be found in Tables 1 and 2. A total of 21 samples were analyzed in this study, including 11 samples that were published^25,26^, with three biological replicates per group. In this study, we generated 201,097,398 raw reads using the Illumina NextSeq. 500 system. After undergoing Illumina quality control, the sequencing reads were subjected to 5′ and 3′-adaptor trimming, and reads with lengths less than 14 nucleotides or greater than 40 nucleotides were discarded using cutadapt. This resulted in a total of 170,323,089 trimmed reads. Among these, 21,607,372 reads were aligned to tRNA sequences, accounting for 12.7% of the total reads (Table 3). Our study successfully characterized the expression profiles of tsRNAs in subcutaneous and omental adipose tissues of lean and obese pigs, as well as in mouse BAT, iWAT, and eWAT. Furthermore, by comparing the expression profiles of pig and mouse tsRNAs, we identified 117 conserved tsRNAs in both pig and mouse adipose tissues. These findings serve as a valuable resource and foundation for further investigation into the epigenetic mechanisms underlying fat deposition.

**Table 1:** Basic sample collection information of Pigs.

Sample	Age (d)	Weight (kg)	Average backfat thickness (mm)	Lean meat percentage (%)
Qingyu1	300	128	60.31	40.39
Qingyu2	300	136.5	42.98	38.89
Qingyu3	300	135	57.36	41.35
Yorkshire1	180	98.5	20.69	54.88
Yorkshire2	180	111	19.2	63.2
Yorkshire3	180	85.5	12.31	61.35

**Table 2:** Basic sample collection information of mice.

Sample	Age (d)	Weight (g)	BAT (g)	iWAT (g)	eWAT (g)
C57BL/6J-1	56	28.1	0.101	0.145	0.525
C57BL/6J-2	56	26.6	0.181	0.104	0.458
C57BL/6J-3	56	28.7	0.137	0.085	0.403

**Table 3:** Mapping summary.

Item	Number
Raw reads	201097398
Trimmed reads	170323089
tsRNAs reads	21607372
tsRNAs reads (%)	12.69

Raw reads: Raw sequencing reads after quality filtering. Trimmed reads: Reads number after 5′, 3′-adaptor trimmed and discarded reads (length < 14nt or > 40nt). tsRNAs reads: Reads number aligned to mature and precursor tRNA. tsRNAs reads(%): The proportion of reads number aligning to mature and precursor tRNA.

## Methods

### Sample collection and RNA extraction

To ensure the animals’ welfare, three adult female Qingyu pigs (obese type), and three adult female Yorkshire pigs (lean type), were humanely euthanized. Following the slaughtering process, the section of subcutaneous fat from the back of the final rib cage, as well as the omental adipose tissue, were promptly excised and immediately submerged in liquid nitrogen for rapid freezing and subsequent storage. Similarly, normal 8-week-old female C57BL/6j mice were humanely sacrificed, and BAT, iWAT and eWAT were collected and frozen in liquid nitrogen. RNA extraction from the subcutaneous adipose tissue and omental adipose tissue, as well as BAT, eWAT and iWAT, was performed using TRIzol reagent (TaKaRa, Japan) according to the manufacturer’s instructions.

### Libraries preparation, sequencing, and tsRNAs mapping

The total RNA samples were assessed for quality using agarose gel electrophoresis and quantified using a Nanodrop spectrophotometer. tsRNAs are heavily decorated by RNA modifications that interfere with small RNA-seq library construction. We do the following treatments before library preparation for total RNA samples: 3′-aminoacyl (charged) deacylation to 3′-OH for 3′adaptor ligation, 3′-cP (2′,3′-cyclic phosphate) removal to 3′-OH for 3′adaptor ligation, 5′-OH (hydroxyl group) phosphorylation to 5′-P for 5′-adaptor ligation, m1A and m3C demethylation for efficient reverse transcription. cDNA was then synthesized and amplified using Illumina’s proprietary RT primers and amplification primers. Subsequently, ~134–160 bp PCR amplified fragments were extracted and purified from the PAGE gel. And finally, the completed libraries were quantified by Agilent 2100 Bioanalyzer. The libraries were denatured and diluted to a loading volume of 1.3 ml and loading concentration of 1.8pM. Raw sequencing data obtained from the Illumina NextSeq. 500 platform underwent a stringent quality control process, which included filtering for Illumina chastity. The trimmed reads, with their 5′ and 3′-adaptor bases removed, were aligned to mature tRNA sequences, allowing for a maximum of 1 mismatch. If any reads failed to align, they were subsequently aligned to precursor tRNA sequences, again permitting only 1 mismatch. Bowtie software was utilized for these alignment processes^27^. Based on a statistical analysis of the alignments, which considered factors such as mapping ratio, read length, and fragment sequence bias, a determination was made regarding the usability of the results. If deemed suitable, expression profiling and the identification of differentially expressed tsRNAs were performed.

### Expression profiling of tsRNAs

The abundance of tsRNAs is assessed by calculating their sequencing counts and normalizing them as counts per million of total aligned reads (CPM). To determine the expression level of each tsRNA, the mapped reads number is utilized and assigned an identification (ID). Subsequently, a filtering step is performed to exclude tsRNAs with a CPM value less than 20 in all samples.The formula for calculating the abundance of tsRNAs is as follows:

$$Count=\mathop{\sum }\limits_{i=1}^{n}\frac{{c}_{i}}{{m}_{i}}$$

i: The i-th read aligned to the tsRNAs region.

n: The number of the reads aligned to the tsRNAs region

c_i_: The count of the i-th read

m_i_: The number of tsRNAs generated from the i-th read (m_i_ possibly occur great than one, only when allowing for more than 1 mismatch).

The CPM value of tsRNAs can be calculated with the formula.

$$CPM=\frac{1{0}^{6}Count}{N}$$

N: The total number of reads mapped onto all of the mature or precursor tRNA.

### Sequence characteristics of tsRNA profiling of subcutaneous white adipose tissue and omental white adipose tissue of pig

**Fig. 2:**
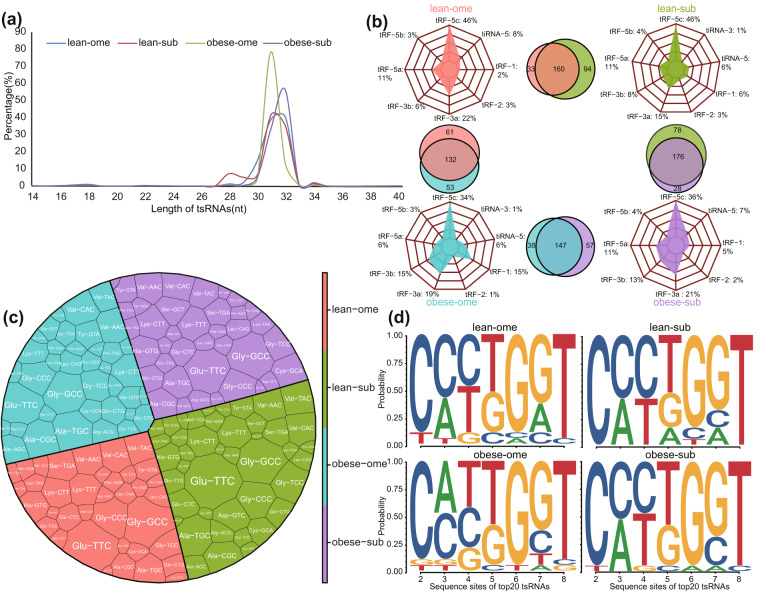
Sequence characteristics of tsRNA profiling of subcutaneous white adipose tissue and omental white adipose tissue of pig. (**a**) Length distribution of tsRNAs. (**b**) Venn diagram and type of identified tsRNAs (CPM > 20). (**c**) Proportion of parental tRNAs of identified tsRNAs. (**d**) Characterization of the seed sequences of four groups of top20 tsRNAs.

Based on the calculated CPM values, we evaluated the tsRNAs expression profile characteristics of subcutaneous white adipose tissue and visceral adipose tissue in lean and obese pigs. We observed the presence of tsRNAs in the subcutaneous adipose tissue of lean pigs (lean-sub), omental adipose tissue of lean pigs (lean-ome), subcutaneous adipose tissue of obese pigs (obese-sub), and omental adipose tissue of obese pigs (obese-ome) within the length range of 14–40 nucleotides. Among these, tsRNAs with a length of approximately 30–33 nucleotides were found to be the most abundant (Fig. 2a). In total, we identified 485 unique tsRNAs across the subcutaneous and omental adipose tissues of lean and obese pigs. A total of 287 tsRNAs were identified in the adipose tissues of lean pigs, with overlapping tsRNAs representing 56.34% of the total. Similarly, 242 tsRNAs were identified in the adipose tissues of obese pigs, with overlapping tsRNAs accounting for 60.74%. In both omental adipose tissue of lean and obese pigs, a total of 246 tsRNAs were found to overlap, constituting 53.66% of the total. Furthermore, in the subcutaneous adipose tissue of lean and obese pigs, 282 tsRNAs were identified, with overlapping tsRNAs accounting for 62.41% (Fig. 2b). Regarding the classification of tsRNAs, we observed that tRF-5c was the most abundant type in all four groups, followed by tRF-3a (Fig. 2b). In the lean-ome group, Glu-TTC, Gly-GCC, and Gly-CCC were the parental tRNAs of tsRNAs with the highest percentage, accounting for 11.38%, 10.16%, and 5.59%, respectively. In the lean-sub group, Glu-TTC, Gly-GCC, and Ala-TGC were the parental tRNAs of tsRNAs with the highest proportion, accounting for 11.75%, 7.83%, and 5.12%, respectively. In the obese-ome and obese-sub groups, Glu-TTC, Gly-GCC, and Gly-GCC were the parental tRNAs of tsRNAs with the highest proportion, accounting for 8.23%, 8.23%, and 7.82%, and 8.85%, 8.85%, and 5%, respectively (Fig. 2c). In addition, we conducted an analysis of nucleotide deviations in the seed sequence positions of the top 20 expressed tsRNAs within each of the four groups. The findings revealed slight variations in the seed sequence bases among the top 20 tsRNAs in the four groups (Fig. 2d).

### Sequence characteristics of tsRNA profiling of BAT, eWAT and iWAT of mice

**Fig. 3:**
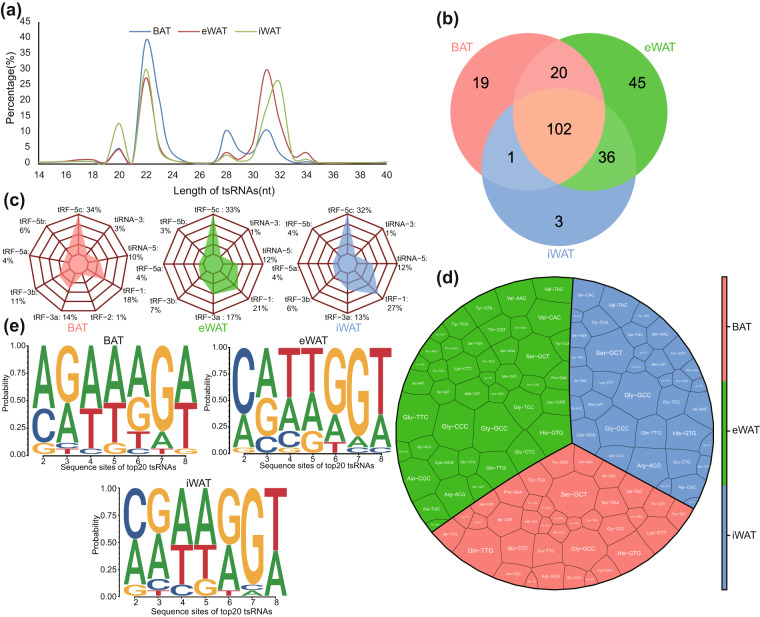
Sequence characteristics of tsRNA profiling of BAT, eWAT and iWAT of mice. (**a**) Length distribution of tsRNAs. (**b**) Venn diagram of identified tsRNAs (CPM > 20). (**c**) type of identified tsRNAs. (**d**) Proportion of parental tRNAs of identified tsRNAs. (**e**) Characterization of the seed sequences of top20 tsRNAs.

Based on the calculated CPM values, we also evaluated the tsRNAs expression profile characteristics of mouse BAT, iWAT, and eWAT. Interestingly, unlike tsRNAs derived from porcine adipose tissue, tsRNAs from mouse adipose tissue showed a predominant length distribution in the ranges of 20–24nt and 28–33nt (Fig. 3a). A total of 278 tsRNAs were identified across the three types of mouse adipose tissues. Among these, we identified 19 BAT-specific tsRNAs, 45 eWAT-specific tsRNAs, and 3 iWAT-specific tsRNAs. The overlapping tsRNAs shared between iWAT and eWAT accounted for 66.67% of their respective expressed tsRNAs. Additionally, the overlapping tsRNAs between BAT and iWAT, as well as between BAT and eWAT, accounted for 56.91% and 52.36% of their expressed tsRNAs, respectively (Fig. 3b). Further analysis of tsRNA types in mouse adipose tissue revealed that, similar to porcine adipose tissue, tRF-5c was the most abundant type of tsRNA. However, unlike porcine adipose tissue, the second most abundant type in mouse adipose tissue was tRF-1 (Fig. 3c). The most common parental tRNAs for tsRNAs were Ser-GCT, Gln-TTG, and Gly-GCC, comprising 8.5%, 7.19%, and 6.54% of the total in the BAT group, respectively. Meanwhile, in the iWAT group, the prevalent parental tRNAs for tsRNAs were Gly-GCC, Ser-GCT, and Ser-GCT, accounting for 8.44%, 7.79%, and 5.84%, respectively. Turning to the eWAT group, the prevailing parental tRNAs for tsRNAs were Gly-GCC, Glu-TTC, and Gly-CCC, accounting for 6.81%, 6.38%, and 5.53% of the total, respectively (Fig. 3d). Additionally, we conducted a similar analysis of the nucleotide deviations in the seed sequence positions of the top 20 expressed tsRNAs in the three mouse adipose tissue groups(Fig. 3e). Interestingly, the nucleotide deviations observed in tsRNAs from mouse adipose tissue and porcine adipose tissue were markedly different.

### Conserved tsRNAs in adipose tissue of pigs and mice

**Fig. 4:**
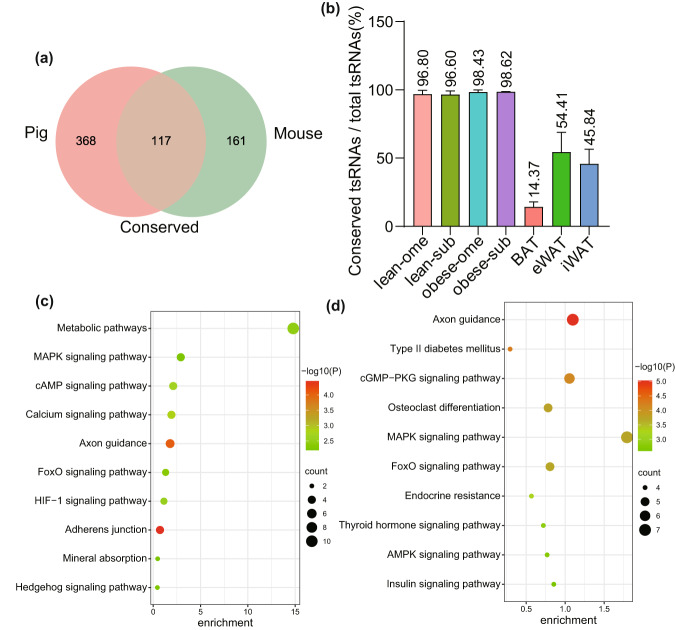
Conserved tsRNAs in adipose tissue of pigs and mice. (**a**) Venn diagram illustrating conserved tsRNAs between porcine and mouse adipose tissues. (**b**) Percentage of conserved tsRNA expression levels in each sample. (**c**) KEGG analysis of potential target genes of tsRNAs derived from Gly-GCC. (**d**) KEGG analysis of potential target genes of tsRNAs derived from Glu-CTC.

By comparing the expression profiles of pig and mouse tsRNAs, we identified 117 tsRNAs that were fully conserved between pigs and mice (Supplementary Table S1), accounting for 18.11% of the total tsRNAs identified (Fig. 4a). Moreover, we analyzed the expression percentage of these conserved tsRNAs in each sample. To our surprise, we found that these conserved tsRNAs were highly expressed, accounting for over 96% of the samples in pigs. However, in mice, the expression percentages of these conserved tsRNAs were relatively lower. In iWAT and eWAT tissues, the expression levels accounted for 45.84% and 54.41%, respectively, while in BAT, it was only 14.37% (Fig. 4b). Interestingly, we found that among the conserved tsRNAs, the TOP10 tsRNAs with the highest average expression abundance were mainly derived from two parental tRNAs, Gly-GCC and Glu-CTC. Among them, there were 7 tsRNAs derived from Gly-GCC and 2 tsRNAs derived from Glu-CTC, and the tsRNAs derived from the same parental tRNA had similar seed sequences (Supplementary Table S1). Therefore, we further predicted their potential target genes on the online prediction website Targetscan (https://www.targetscan.org/vert_50/seedmatch.html), and performed Kyoto Encyclopedia of Genes and Genomes (KEGG) enrichment analysis on the online tool KOBAS (http://bioinfo.org/kobas). KEGG analysis showed that the potential target genes of tsRNA derived from Gly-GCC were mainly enriched in signal transduction-related pathways, such as Calcium signaling pathway and cAMP signaling pathway, and metabolism-related pathways, such as Metabolic pathways, FoxO signaling pathway and MAPK signaling pathway (Fig. 4c). The potential target genes of tsRNA derived from Glu-CTC were mainly enriched in obesity disease-related pathways, such as Type II diabetes mellitus, and metabolic-related pathways, such as Insulin signaling pathway, MAPK signaling pathway, FoxO signaling pathway, Thyroid hormone signaling pathway and AMPK signaling pathway (Fig. 4d).

## Data Records

The tsRNA sequencing data for the 12 porcine adipose tissue samples and 9 mouse adipose tissue samples referred to in this article have been uploaded to the NCBI Sequence Read Archive (SRA) under access number SRP407032^28^ and SRP439993^29^. The expression profile of tsRNAs in pigs can be obtained in the Gene Expression Omnibus (GEO) database of the NCBI, and the assession number is GSE235087^30^. The expression profile of mouse tsRNAs can also be obtained in the GEO database, and the assession number is GSE235088^31^.

## Technical Validation

### Sequencing quality control

**Fig. 5:**
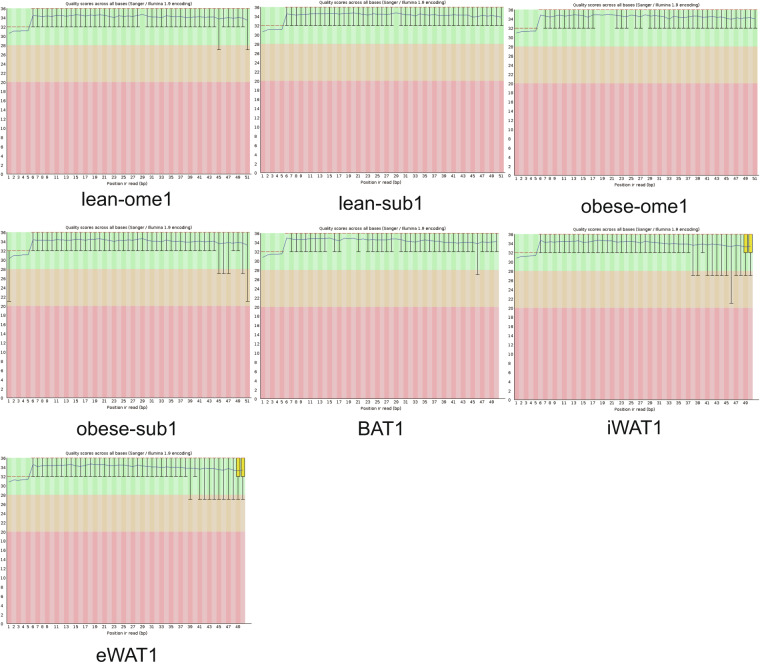
tsRNA-seq quality score plot. The X-axis displays the position in the read, while the Y-axis represents the Q value. The median Q score is shown as a red line, and the mean Q score is depicted as a blue line. The inter-quartile range is represented by the boxplot, and the whiskers extend to the 10% and 90% points. Data with a Q score exceeding 30, indicating accuracy above 99.9%, is considered high-quality.

Raw data files in FASTQ format were obtained from the Illumina sequencer. To assess the quality of the sequencing data, we performed quality control using FastQC (http://www.bioinformatics.babraham.ac.uk/projects/fastqc/). We generated plots displaying the quality scores for each sample (Fig. 5; Supplementary Figures S1). The quality score, denoted as Q, is logarithmically associated with the probability of base calling errors (*P*).

$$Q=-1{0\log }_{10}\left(P\right)$$

For example, Q30 means the incorrect base calling probability to be 0.001 or 99.9% base calling accuracy. All samples of Q30 are shown in Table 4.

**Table 4:** Quality score of the sample used in our study.

Sample	TotalRead	TotalBase	BaseQ. 30	BaseQ. 30 (%)
obese-sub1	11833544	603510744	567352963	94.01
obese-sub2	8256383	421075533	391482779	92.97
obese-sub3	10399443	530371593	493002831	92.95
obese-ome1	8079564	412057764	379340758	92.06
obese-ome2	9529406	485999706	452234929	93.05
obese-ome3	10168082	518572182	482763022	93.09
lean-sub1	11373476	580047276	537160780	92.61
lean-sub2	11199763	571187913	532242409	93.18
lean-sub3	12980769	662019219	609990267	92.14
lean-ome1	13108635	668540385	622927286	93.18
lean-ome2	12112953	617760603	575238627	93.12
lean-ome3	8544746	435782046	400493079	91.90
BAT1	10395534	519776700	486707550	93.64
BAT2	8114960	405748000	371737506	91.62
BAT3	8898480	444924000	410684324	92.30
eWAT1	7084165	354208250	326496003	92.18
eWAT2	7755765	387788250	357414689	92.17
eWAT3	5759943	287997150	280278763	97.32
iWAT1	7835103	391755150	360597465	92.05
iWAT2	8814977	440748850	404587758	91.80
iWAT3	8851707	442585350	405879110	91.71

Sample: Sample ID. TotalRead: Total sequencing reads post quality filtering. TotalBase: Total bases post quality filtering. BaseQ. 30: Bases with Q score greater than 30 post quality filtering. BaseQ. 30 (%): Percentage of bases (Q ≥ 30) post quality filtering.

### Reproducibility validation

**Fig. 6:**
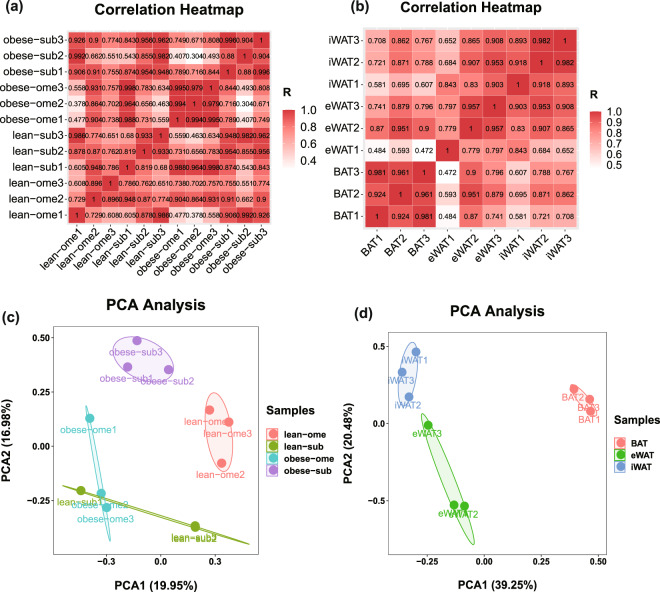
Assessment of reproducibility across biological replicates. (**a,b**) Heatmap of correlation of pig and mice samples respectively. (**c,d**) PCA analysis of pig and mice samples respectively.

To assess the reproducibility of the biological replicates in our sample set, we conducted correlation analysis on the 12 samples obtained from pigs and mice. The correlation heatmap revealed high correlation coefficients among the majority of biological replicates (Fig. 6a,b). Additionally, principal component analysis (PCA) demonstrated that most of the biological samples clustered together (Fig. 6c,d). These results provide strong evidence for the high confidence and reliability of our study data.

### Supplementary information

Supplementary Table S1

Supplementary Information

## Data Availability

All software used in this study species are in the public domain, except for those explicitly described in the text and methods. No custom scripts or code was used during the curation and validation of the dataset in this study. Also the following software tools were used: tRNA sequences of cytoplasmic were downloaded from GtRNAdb^32,33^. tRNA sequences of mitochondrial were predicted with tRNAscan-SE^34,35^ software. Principal Component Analysis (PCA), Correlation Analysis, Pie plots, Venn plots, and radar plots were performed in the online analysis tool Bioinformatics (www.bioinformatics.com). The seed sequence motif was done using the BioLadder online tool (www.bioladder.cn/).
