# Supplementary material for: Dynamic changes in the transcriptome of tRNA-derived small RNAs related with fat metabolism
Source: Sci Data. 2023 Oct 14;10:703. doi: 10.1038/s41597-023-02624-y (PMC10576826; doi:10.1038/s41597-023-02624-y)
Supplement: Supplementary file 2 — Supplementary Information [file 41597_2023_2624_MOESM2_ESM.pdf]

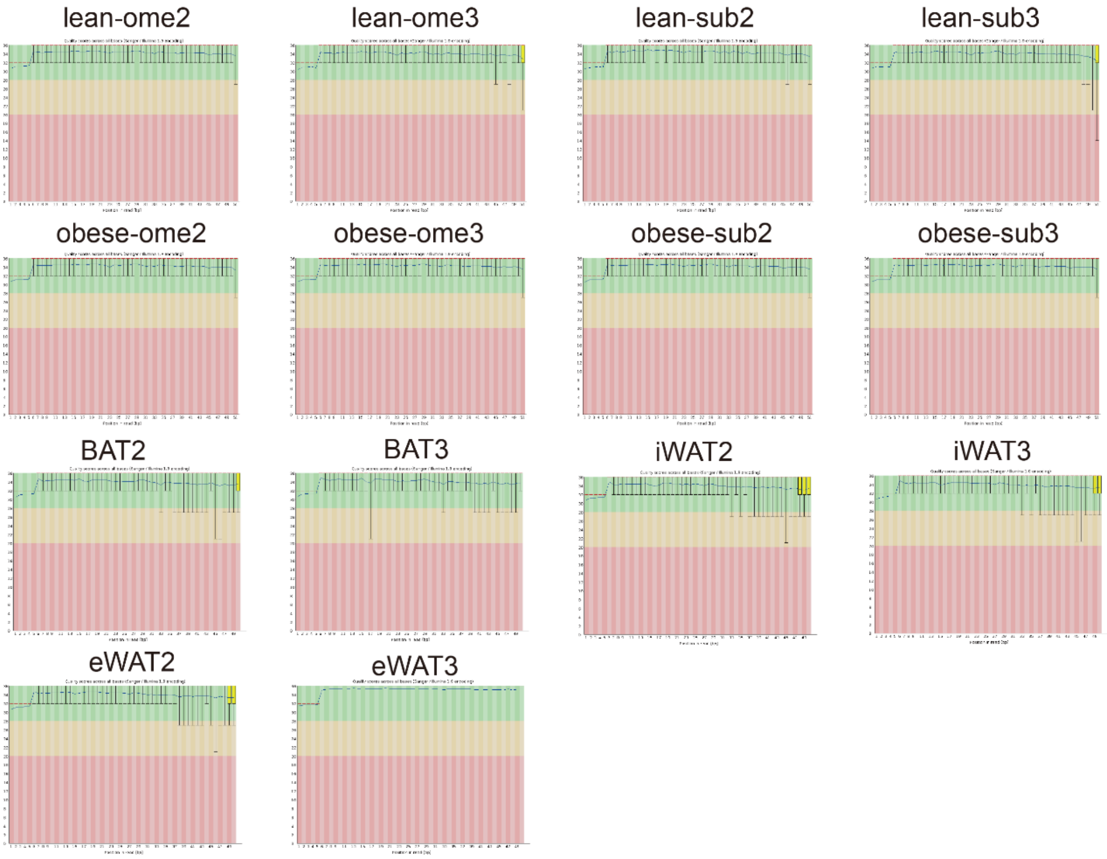

Supplementary Figures S1. All tsRNA-seq quality score plot. The position in the read is plotted on the X-axis and the Q value is plotted on the Y-axis. The red line is the median Q score, and the blue line is the mean Q score. The boxplot represents the inter-quartile range, while the whiskers represent the 10% and 90% points. A Q score above 30 (>99.9% correct) is considered high quality data
